# Direct-to-Consumer Educational Brochures to Promote Gabapentinoid Deprescribing in Older Adults

**DOI:** 10.1001/jamainternmed.2024.4748

**Published:** 2024-09-23

**Authors:** Marc-Alexandre Gingras, Robert Dubé, Jerome Williams, James Shih, Anthony Lieu, Tania Morin, Kristen Moran, Iman Huseen, Todd C. Lee, Emily G. McDonald

**Affiliations:** 1 Division of General Internal Medicine, Department of Medicine, Sherbrooke University, Sherbrooke, Quebec, Canada; 2 Faculty of Medicine and Health Sciences, McGill University, Montreal, Canada; 3 Clinical Practice Assessment Unit, Department of Medicine, McGill University Health Centre, Montreal, Quebec, Canada; 4 Division of General Internal Medicine, Department of Medicine, McGill University Health Centre, Montreal, Quebec, Canada; 5 The Canadian Medication Appropriateness and Deprescribing Network, Montreal, Quebec, Canada

## Abstract

This study examines gabapentinoid deprescribing using a novel approach of direct-to-consumer educational brochures intended to empower older adults to initiate a discussion on gabapentinoid risks and tapering.

Systematic reviews of randomized clinical trials do not support the off-label use of gabapentinoids for pain because the harms often outweigh the benefits.^1,2^ Nonetheless, gabapentinoid prescription rates continue to climb.^3^ Deprescribing gabapentinoids represents an opportunity to improve care, particularly among vulnerable older adults. The primary aim of this clinical trial was to increase gabapentinoid deprescribing using a novel approach of direct-to-consumer educational brochures intended to empower older adults to initiate a discussion on gabapentinoid risks and tapering with their clinican.^4^

## Methods

This prospective before-and-after study (eFigure in Supplement 1) took place at the McGill University Health Centre; the trial protocol has been published,^5^ with additional details in Supplement 2 and the eMethods in Supplement 1. Transparent Reporting of Evaluations with Nonrandomized Designs (TREND) guidelines were followed. Patient consent was obtained, and ethics approval was granted by McGill University Health Centre Research Ethics Board.

Patients 60 years and older receiving a gabapentinoid at the time of hospitalization for any indication other than seizure were eligible. Patients initially enrolled in the study received usual care. During the intervention phase, patients received a direct-to-consumer educational brochure^4^ informing on the potential risks of gabapentinoids, nonpharmacologic alternatives, and a proposed deprescribing regimen. This was coupled with monthly gabapentinoid educational sessions for clinicians on the study wards.^5^

The primary outcome was gabapentinoid deprescription at 8 weeks after discharge (cessation or ongoing taper with intent to stop). Secondary outcomes included changes to global health, pain, and cognition (captured through Patient-Reported Outcomes Measurement Information Systems [PROMIS]^6^ questionnaires), initiation/changes to other pain medications, and gabapentinoid dose reductions without intention to stop. Outcomes were collected via a postdischarge telephone interview and validated using the provincial drug database. Analysis was according to intention-to-treat, with binary outcomes assessed using binomial regression, comparing intervention and usual care, adjusting a priori for sex and age. Raw patient-reported outcome scores were converted to T scores and compared between intervention and usual care by linear regression adjusting for age, sex, and baseline T score. All analyses were performed with STATA version 17 (Statacorp).

## Results

Enrollment of this 160-participant trial began May 2021 with 15 months of usual care (n = 80) followed by 13 months of intervention (n = 80). Overall, 80 of 160 patients (50.6%) were female and 133 (83.1%) were prescribed pregabalin. Common off-label indications were for pain (32.5%; n = 52) and osteoarthritis (25.6%; n = 41) (Table 1). In each group, 9 patients (11.3%) did not complete the study (eFigure 1 in Supplement 1); there were 13 deaths (7 in the usual care group and 6 in the intervention group) unrelated to the intervention. Among those who completed the study, deprescription occurred in 7 of 71 patients (9.9%) in the usual care group vs 15 of 71 patients (21.1%) in the intervention group, corresponding to an age- and sex-adjusted absolute increase in deprescribing of 13.3% (95% CI, 2.0% to 24.6%; number needed to treat, 8) (Table 2). Doses of concurrent pain medications did not increase and new pain medications were not initiated. There were no significant differences in PROMIS score changes at follow-up (T score differences: global health, −0.8 [95% CI, −3.0 to 1.3]; pain, −2.5 [95% CI, −5.8 to 0.8]; cognition 1.8 [95% CI, −1.1 to 4.7]).

**Table 1.** Patient Characteristics

Characteristic	No. (%)	
	Usual care (n = 80)	Intervention (n = 80)
Demographics		
Age, median (IQR), y	76 (68-83.5)	73 (66-81)
Female	43 (53.8)	38 (47.5)
Male	37 (46.3)	42 (52.5)
Independent living	72 (90.0)	64 (80.0)
Semi-autonomous living	5 (6.3)	12 (15.0)
Assisted living or nursing home	3 (3.8)	4 (5.0)
Length of stay, median (IQR), d	10 (7-20)	11 (6-26.5)
Admission diagnosis (top 5)		
Congestive heart failure	6 (7.5)	10 (12.5)
Falls or trauma	9 (11.3)	6 (7.5)
Pneumonia	5 (6.3)	9 (11.3)
Cancer	7 (8.8)	6 (7.5)
Skin or bone infection	5 (6.3)	8 (10.0)
Medications		
Gabapentin	14 (17.5)	13 (16.3)
Total daily dose, median (IQR), mg	550 (300-600)	600 (600-600)
Pregabalin	66 (82.5)	67 (83.8)
Total daily dose, median (IQR), mg	100 (50-200)	125 (75-225)
Acetaminophen	70 (87.5)	58 (72.5)
Cannabinoids	4 (5.0)	0
Nonsteroidal anti-inflammatory drugs	13 (16.3)	7 (8.8)
Opiates	36 (45.0)	22 (27.5)
Tramadol	3 (3.8)	1 (1.3)
Comorbidity		
Asthma or chronic obstructive pulmonary disease	26 (32.5)	25 (31.3)
Atrial fibrillation	25 (31.3)	21 (26.3)
Cancer	18 (22.5)	20 (25.0)
Chronic kidney disease	20 (25.0)	14 (17.5)
Congestive heart failure	8 (10.0)	14 (17.5)
Diabetes	23 (28.8)	28 (35.0)
Hypertension	58 (72.5)	61 (76.3)
Ischemic heart disease	24 (30.0)	26 (32.5)
Ischemic stroke	13 (16.3)	11 (13.8)
Potential gabapentinoid indication^a^		
Cancer pain	12 (15.0)	7 (8.8)
Diabetic neuropathy	16 (20.0)	15 (18.8)
Fibromyalgia	3 (3.8)	4 (5.0)
Osteoarthritis pain	23 (28.8)	18 (22.5)
Other neuropathy	3 (3.8)	7 (8.8)
Other pain	25 (31.3)	27 (33.8)
Pain related to a trauma	17 (21.3)	9 (11.3)
Postherpetic neuralgia	1 (1.3)	0
Sciatica	3 (3.8)	5 (6.3)

^a^ Patient could have had more than 1 indication.

**Table 2.** Patients with Gabapentinoid Deprescribed and Mean Change in Patient-Reported Outcomes by Intervention Status

Outcome	Usual Care (n = 71)	Intervention (n = 71)	Risk difference (95% CI), %^a^
Primary outcome at 8 wk, No. (%)			
Gabapentinoid deprescription	7 (9.9)	15 (21.1)	13.3 (2.0 to 24.6)
Secondary outcomes at 8 wk, No. (%)			
Gabapentinoid dose reduction without intention to stop	9 (12.7)	9 (12.7)	2.5 (−9.3 to 14.3)
New pain medicine prescribed	10 (14.1)	12 (16.9)	1.5 (−9.1 to 12.0)
Existing pain medicine increased	6 (8.5)	1 (1.4)	−7.0 (−14.1 to 0.01)^b^
Participant-reported outcomes, mean (SD)			Difference (95% CI)^c^
Global physical health			
Baseline T score	33.2 (7.2)	33.2 (7.5)	
8-wk T score	34.1 (7.4)	33.4 (6.5)	−0.8 (−3.0 to 1.3)
Pain intensity			
Baseline T score	50.3 (10.9)	51.2 (10.3)	
8-wk T score	49.1 (12.3)	47.8 (10.8)	−2.5 (−5.8 to 0.8)
Cognitive function			
Baseline T score	31.1 (8.4)	33.1 (9.3)	
8-wk T score	29.5 (8.2)	32.7 (9.8)	1.8 (−1.1 to 4.7)

^a^ Adjusted for age and sex using binomial regression comparing intervention vs usual care groups.

^b^ Did not converge with model, presented unadjusted.

^c^ Adjusted for age, sex, and baseline T score using linear regression comparing intervention vs usual care groups.

## Discussion

Results of this single-center pilot trial support the use of direct-to-consumer educational brochures for deprescribing gabapentinoids in hospitalized older adults and warrant further study in a multicenter cluster randomized trial. The major limitation was the before-and-after, single-site, unblinded study design, without a contemporaneous control. The unblinded design could have rendered clinicians susceptible to the Hawthorne effect and patients susceptible to a social desirability bias. Changes due to temporal trends couldn’t be ascertained, although implementing the intervention at 15 months may have limited variations secondary to trainee experience. The short duration of follow-up also limits conclusions about the durability of the intervention. Major strengths were the novel approach leveraging brochures that are free of charge,^4^ scalable, and foster patient self-empowerment, and the collection of patient-reported outcomes, often missing from deprescribing trials, examining the impact of the intervention on pain, global health, and cognition.

## Notes

Supplement 1. eMethods

Supplement 2. Trial protocol

Supplement 3. Data sharing statement
