# Supplementary material for: Direct-to-Consumer Educational Brochures to Promote Gabapentinoid Deprescribing in Older Adults
Source: JAMA Intern Med. 2024 Sep 23;184(11):1386–8. doi: 10.1001/jamainternmed.2024.4748 (PMC11420817; doi:10.1001/jamainternmed.2024.4748)
Supplement: Supplement 1. — eMethods [file jamainternmed-e244748-s001.pdf]

Supplemental Online Content

Gingras M, Dubé R, Williams J, et al. Direct-to-Consumer Educational Brochures to Promote Gabapentinoid Deprescribing in Older Adults. *JAMA Intern Med.* Published online September 23, 2024. doi:10.1001/jamainternmed.2024.4748

eMethods 2

Study design and setting 2

Participants and Inclusion/exclusion criteria 2

Trial interventions 3

Data Collection 4

Primary and Secondary Outcomes 4

Sample Size 5

Statistical Analysis 5

Ethics 5

eFigure: CONSORT Flow Diagram of Older Adults Admitted to the Hospital and Prescribed Gabapentinoids at Home 6

References: 6

This supplemental material has been provided by the authors to give readers additional information about their work.

© 2024 American Medical Association. All rights reserved.

eMethods

Study design and setting

This prospective before-and-after non-randomized controlled study took place on five medical units of the McGill University Health Centre, comprising the Royal Victoria Hospital and Montreal General Hospital. The primary aim of the study was to increase deprescription of gabapentinoids using new educational brochures designed in collaboration with the Canadian Medication Appropriateness and Deprescribing Network (CADeN). The detailed study protocol has been previously published[1].

This was a trainee-led project which was funded using internal quality improvement funds. A cluster randomized trial was not possible as this would require more clusters and a larger sample size than we had access to with our two hospitals. A multicenter cluster randomized RCT would have been more ideal, but this was outside the scope of the budget and timelines of this study.

Participants and Inclusion/exclusion criteria

Recruitment for this study began in May 2021. We screened all patients admitted to the participating medical units for active chronic home gabapentinoid use, identified on admission medication reconciliation performed by the unit pharmacists, as part of standard of care. All medications that patients are taking on the medical unit upon admission to our hospital undergo a process of medication reconciliation whereby a pharmacist verifies with the patient, family, and/or community pharmacy that the prescription is active and being taken by the patient. Chronic gabapentinoid use was defined as ongoing use predating admission, regardless of the start date and without a formal requirement for the minimum duration.

© 2024 American Medical Association. All rights reserved.

Those aged 60 and above were eligible for this study given their higher risk for polypharmacy and adverse drug events.[2] We excluded patients if they were not enrolled in the provincial public health insurance plan and those with a known seizure disorder (given an anti-epileptic effect of gabapentinoids); a life expectancy less than 3 months; the inability to consent; a major neurocognitive disorder; and/or the inability to read English or French.

Trial interventions

All sites began in the usual care period of the trial, in which patients received usual medical care. Participants were informed that the general aims of the study were to evaluate usual home pain medications and prescribing trends at discharge, without specifically mentioning gabapentinoids. Similarly, healthcare workers were unaware of the specific class of medications being studied.

Once 80 patients were enrolled into the usual care arm, all five medical units transitioned to the intervention period of the trial. The intervention consisted of 2 components. To begin, after consenting to our trial, patients received the direct-to-consumer educational brochure[3], consisting of information and statistics on the risks of gabapentinoids, non-pharmacologic therapeutic alternatives, and a proposed tapering regimen. Secondly, an educational session about gabapentinoids, the purpose of our study, and the brochures themselves, were introduced to all physicians and medical students delivering care on the study units (previously described)[1]. The educational intervention was delivered at the start of each 4-week rotation block, whereas patients consented to the study continuously.

© 2024 American Medical Association. All rights reserved.

Data Collection

All participants provided written consent. Our research assistants used pharmacy records, patient charts, and a comprehensive questionnaire to gather all demographic information and complete medical histories. We also assessed global health[4], pain control[5], and cognition[6] using the Patient-Reported Outcomes Measurement Information Systems (PROMIS) Questionnaires administered in the patient's language of choice (French or English).

Patients were telephoned 8 weeks following hospital discharge, during which time we performed our follow up questionnaire to track changes in medical issues, ongoing deprescribing of gabapentinoids, and other changes (including the addition) of other pain medications. We also repeated the PROMIS questionnaires.

Primary and Secondary Outcomes

The primary outcome was the deprescription of gabapentinoids at 8 weeks post hospital discharge, defined as complete cessation or an ongoing taper with explicit intention to discontinue (meaning a taper was prescribed ending in a completed discontinuation, as opposed to ending in a dose reduction). Cessation was defined by a taper to stop or a prescription to stop, and the absence of dispensation from the patient's community pharmacy, validated after completion of patient questionnaires at 8-weeks. Secondary outcomes included dose reductions in gabapentinoids without intent to stop, the initiation or dose changes of other pain medications, and changes to the patient reported outcomes (e.g., global physical health[4], pain[5], cognition[6]).

© 2024 American Medical Association. All rights reserved.

Sample Size

A sample size of 160 was chosen due to an expected usual care period event rate (deprescription at 8 weeks post discharge) of 13%.[7] This sample size would allow for detection of an absolute increase of 20% in deprescription rates, allowing for a 2-sided type 1 error of 5% and a type 2 error of 20%, when accounting for a projected 15% loss to follow up. Previous trials have demonstrated an absolute increase of 43% in benzodiazepine deprescription in similar inpatient settings[8], thus a rate of 20% was felt to be a conservative estimate for our trial.

Statistical Analysis

An intention-to-treat analysis was performed for this study. We assessed binary outcomes using binomial regression, comparing intervention and usual care groups, adjusting *a priori* for sex and age. For the patient reported outcomes, the raw scores were converted to T-scores using the appropriate scoring tool and a linear regression was performed adjusting for the age, sex, and baseline T-score. All analyses were performed using STATA version 17 (StataCorp LP, USA).

Ethics

Ethics approval was granted by the McGill University Health Centre Research Ethics Board.

© 2024 American Medical Association. All rights reserved.

eFigure: CONSORT Flow Diagram of Older Adults Admitted to the Hospital and Prescribed Gabapentinoids at Home

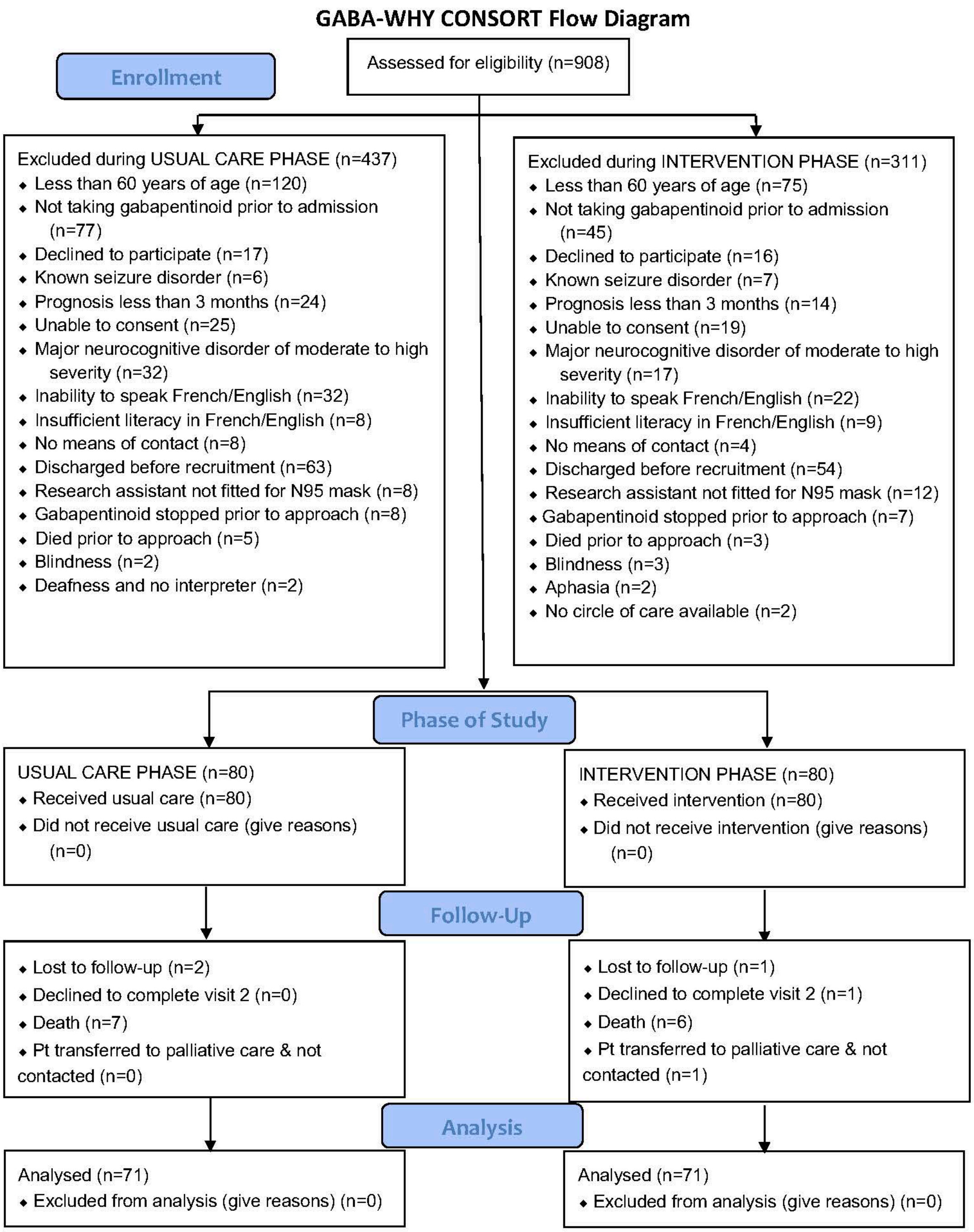

© 2024 American Medical Association. All rights reserved.

References:

1. Williams J, Gingras MA, Dubé R, Lee TC, McDonald EG. Patient empowerment brochures to increase gabapentinoid deprescribing: protocol for the prospective, controlled before-and-after GABA-WHY trial. *CMAJ Open*. 2022;10(3):E652-E656. doi:10.9778/cmajo.20210302

2. Ross MSc Candidate SB, Wu PE, Atique Md Candidate A, et al. Adverse Drug Events in Older Adults: Review of Adjudication Methods in Deprescribing Studies. *J Am Geriatr Soc*. 2020;68(7):1594-1602. doi:10.1111/jgs.16382

3. You may be at risk: Gabapentinoids. Accessed April 22, 2024. https://static1.squarespace.com/static/5836f01fe6f2e1fa62c11f08/t/63b6f36e4075fc54e43fce17/1672934258831/Gabapentinoids_EN_Brochure.pdf

4. Hays RD, Schalet BD, Spritzer KL, Cella D. Two-item PROMIS® global physical and mental health scales. *J Patient-Rep Outcomes*. 2017;1:2. doi:10.1186/s41687-017-0003-8

5. Module: PROMIS Pain Intensity - Short Form 3a v1.0 | NIDA CTN Common Data Elements. Accessed April 22, 2024. https://cde.nida.nih.gov/instrument/0a481bfb-a5e6-3c84-e050-bb89ad43314d/module/0a481bfb-a5e9-3c84-e050-bb89ad43314d

6. PROMIS Short Form v2.0 - Cognitive Function 6a. Accessed August 15, 2021. https://www.healthmeasures.net/index.php?option=com_instruments&view=measure&id=768&Itemid=992

7. Gingras M, Lieu A, Papillon-Ferland L, Lee TC, McDonald EG. Retrospective Cohort Study of the Prevalence of Off-label Gabapentinoid Prescriptions in Hospitalized Medical Patients. *J Hosp Med*. 2019;14(9):547-550. doi:10.12788/jhm.3203

© 2024 American Medical Association. All rights reserved.

8. Torrance N, Veluchamy A, Zhou Y, et al. Trends in gabapentinoid prescribing, co-prescribing of opioids and benzodiazepines, and associated deaths in Scotland. *Br J Anaesth*. 2020;125(2):159-167. doi:10.1016/j.bja.2020.05.017

© 2024 American Medical Association. All rights reserved.
