# Supplementary material for: Direct-to-Consumer Educational Brochures to Promote Gabapentinoid Deprescribing in Older Adults
Source: JAMA Intern Med. 2024 Sep 23;184(11):1386–8. doi: 10.1001/jamainternmed.2024.4748 (PMC11420817; doi:10.1001/jamainternmed.2024.4748)
Supplement: Supplement 2. — Trial protocol [file jamainternmed-e244748-s002.pdf]

**TITLE OF PROPOSAL:**

**GABA-WHY Study:** Deprescription of gabapentinoids in medical inpatients

**Protocol number:** 2021-7353

**Principal investigator:** Emily McDonald, MDCM MSc
**Co-investigators:** Todd Campbell Lee, MD MPH; Marc-Alexandre Gingras, MD MSc

## Lay abstract

Gabapentinoids (gabapentin and pregabalin) are antiepileptics that are frequently prescribed for multiple conditions associated with chronic pain. Current FDA and Health Canada approved indications include painful diabetic neuropathy, postherpetic neuralgia, fibromyalgia and neuropathic pain associated with spinal cord injury [1-4]. However, the majority of current prescriptions are for off-label indications, despite limited proven efficacy or evidence of lack of efficacy [5]. Side effects are common at recommended therapeutic doses and include lower extremity edema, drowsiness, increased risk of traumatic falls, as well as increased risk of death when co-prescribed with opioids[6, 7].

A previously published descriptive study has shown that gabapentinoid use is very common in medical inpatients (1 in 8 admissions) [8]. Only a minority of users (17%) present an approved indication. Many are taking the drug for reasons which have been disproven in randomized controlled trials. Furthermore, gabapentinoid users are more likely to be co-prescribed opioids, benzodiazepines or other sedative drugs which increases the risk of serious harm including death [6, 7].

Polypharmacy, defined as taking 5 or more medications, is extremely common in patients admitted to medical wards. Hospitalizations represent ideal opportunities to review with patients their medications, including initial indications, efficacy and side-effects, with identification of medications that should be appropriately discontinued, or “deprescribed”. Unfortunately, attempts for deprescription of gabapentinoids are seldom made during hospital admissions to medical wards [8].

Different strategies exist to improve deprescription of potentially inappropriate medications during hospitalization. These include direct patient education through the distribution of patient-friendly brochures. These have been shown to lead to increased deprescription rates of sedative hypnotics up to 30 days post-discharge [9].

This trial will evaluate the impact of educational brochures, distributed to inpatients on medical wards, on deprescription rates of gabapentinoids. This intervention will be supplemented by brief information sessions for medical staff. This prospective controlled before-after trial will study the effectiveness of this combined intervention in five medical units, spread across two tertiary-care hospitals in Montréal, Canada.

## Objectives of the research and relevance to safe medical care

Gabapentinoid prescriptions have soared in North America over the past decade. In 2016, pregabalin (brand name Lyrica®) was the eighth medication in the United States with highest invoice spending, with 4.4 billion US dollars spent [10]. This surge in popularity is largely driven by rampant off-label use for various chronic pain conditions, such as osteoarthritis, chronic lower back pain, sciatica and cancer-associated pain [5]. Although there exists reasonable quality evidence to support the use of gabapentinoids for diabetic neuropathy and postherpetic neuralgia, there is currently no evidence or evidence of lack of efficacy for other indications, including various types of neuropathic pain [11-16]. However, in the face of the ongoing opioid crisis, physicians are turning to alternative analgesics, making gabapentinoids a popular choice.

Although serious adverse drug events are rare with gabapentinoids, side effects are common, especially at doses proven to be effective in clinical trials. Common symptoms include sedation, lower

extremity edema and traumatic falls in older adults [17, 18]. Gabapentinoid users are more likely to suffer from chronic cardiac, pulmonary and renal diseases, placing them at higher risk for adverse events [8]. The use of gabapentinoids for their analgesic effects leads to frequent co-prescription with opioids, which increases dramatically the risk of opioid-related death [6, 7]. Furthermore, there is growing concern about abuse and misuse of gabapentinoids, as symptoms of euphoria have been reported with supratherapeutic doses [19]. Several jurisdictions have changed the gabapentinoids to "controlled" status, treating them like narcotics and other drugs with abuse potential.

The common off-label use of gabapentinoids, for which there is no evidence to support efficacy, combined with the high potential for side effects, make this class of medication an ideal target for deprescription. Furthermore, most patients receive total daily doses inferior to those shown to be effective in clinical trials, which can be explained by poor tolerability of the drugs or decreased clearance from renal failure [8]. Unfortunately, deprescription rates of gabapentinoids are very low on medical wards [8].

Distribution of educational brochures to hospitalized patients has been shown to be effective in improving successful deprescription of potentially inappropriate medications [9]. The brochures are standardized 8 to 10-page letter size documents, written in a sixth-grade level vocabulary for ease of comprehension. Examples are available on the Canadian Deprescribing Network website. Specific documents exist for different classes of potentially inappropriate medications, such as sedative-hypnotics, opioids, gabapentinoids and proton pump inhibitors. They contain self-assessment tests on risks of medications, provide information on evidence of efficacy and common side effects, propose therapeutic alternatives and suggest tapering regimens in the form of illustrated calendars. Patients are clearly informed to contact their healthcare providers prior to discontinuing or tapering their medication. Distribution of these educational brochures during hospitalization in chronic sedative users lead to a discontinuation rate of 64% at 30 days post-discharge, versus 21% with usual care [9].

In this trial, we aim to demonstrate that distribution of educational brochures during hospitalization to chronic gabapentinoid users can lead to improvement of successful deprescription. We hope that the brochures will inform patients of the limited evidence to support use of gabapentinoids and the risks of common side effects, motivating them to attempt discontinuation. As clearly stipulated in the brochures, the patients are expected to reach out to a healthcare provider to discontinue the drug, as gabapentinoids need to be gradually tapered to prevent rebound seizures, even in the absence of an underlying seizure disorder. These healthcare providers can either be staff or residents on the medical ward at the time of hospitalization, or a family physician in the community after discharge. By improving deprescription of gabapentinoids, we hope to reduce off-label use and, consequently, unnecessary exposure to potential undesirable side effects, leading ultimately to improved quality of life.

**Our primary objective is to facilitate the deprescription of gabapentinoids using a patient educational brochure.**

Specifically, we aim to:

1. **Primary outcome: evaluate the effectiveness of the patient empowerment intervention tool for deprescription of gabapentinoids in a hospital-based setting** as measured by the proportion of patient with a gabapentinoid prescription deprescribed (stopped or with an intent to taper to discontinuation) at 8-weeks post-hospital discharge.

   This patient empowerment tool is a 10-page brochure based on social constructivist learning and self-efficacy theory. This will be paired with brief monthly orientation sessions for the medical staff and residents of the participating clinical teaching units during the intervention phase. These sessions will review the evidence for efficacy of gabapentinoids and introduce the use of educational pamphlets. Our primary endpoints will be sustained cessation of pre-hospital use at 8 weeks post-discharge. Discontinuation will be confirmed with consent to access to the *Dossier Santé Québec* (DSQ) and, as necessary to contact the respective community pharmacy of the participants.

2. **Secondary outcomes:**
    a. **Dose reduction (without intention to discontinue) measured at 8-weeks post hospital discharge**
    b. **Patient-centered outcomes (measured at 8-weeks post hospital discharge)**:
        i. changes in global functioning scores
        ii. pain control
        iii. cognition

       These outcomes will be evaluated with the use of standardized questionnaires
    c. **Initiation of new pain medications at 8-weeks post-hospital discharge**
    d. **Increases in doses of current pain medications** because of gabapentinoid cessation measured at 8-weeks post hospital discharge

## Background

Gabapentinoids are a class of antiepileptic drugs which includes gabapentin (brand name Neurontin®) and pregabalin (brand name Lyrica®). The original gabapentinoid, gabapentin, was released in the 1990s for treatment of seizures, with pregabalin available a decade later for the same indication [5]. They both bind voltage-gated calcium channels in the central nervous system, inhibiting neuronal calcium influx and consequently release of excitatory neurotransmitters. This mechanism of action was felt to have potential analgesic properties for the treatment of neuropathic pain. Since their release, the FDA and Health Canada have approved the use of gabapentin and pregabalin for treatment of postherpetic neuralgia, with additional indications for pregabalin including painful diabetic neuropathy, fibromyalgia and neuropathic pain associated with spinal cord injury [1-4].

Despite a short list of approved indications, gabapentinoid prescriptions have soared in the past decade in both the United States and Canada [20, 21]. In 2016, Lyrica® was the eighth medication in the United States with highest invoice spending, with 4.4 billion US dollars spent [10]. The rise in gabapentinoid prescriptions has been largely driven by its new-found popularity for various off-label indications related to chronic pain, such as osteoarthritis, chronic lower back pain, sciatica and cancer-related pain [22]. Treating physicians have turned to gabapentinoids as a more acceptable therapeutic alternative to opioids. A Cochrane systematic review published in 2013 found reasonably good evidence for the use of both gabapentin and pregabalin for postherpetic neuralgia and painful diabetic neuropathy, while there was only evidence supporting pregabalin for the treatment of fibromyalgia and central neuropathic pain [16]. Numbers needed to treat varied from 4 to 10 for pain intensity reduction of at least 50% from baseline. However, benefits were compounded by a high risk of adverse events, leading to frequent drug discontinuation. Common side effects of gabapentinoids include sedation, dry mouth, lower extremity edema and traumatic falls in older adults [17, 18]. The poor tolerability of these medications at therapeutic doses might explain why up to half of users receive total daily doses inferior to FDA recommendations [8]. With regards to the treatment of other chronic pain conditions, including various types of neuropathic pain, there is either a lack of evidence or evidence of lack of efficacy to support the use of gabapentinoids [11-15].

These is a high prevalence of gabapentinoid use in patients admitted to medical wards [8]. The multiple comorbidities and rampant polypharmacy among these individuals place them at risk for adverse drug events, justifying careful screening for potentially inappropriate medications upon admission. A recently published retrospective descriptive study found that 1 in 8 patients admitted to a medical ward at a tertiary-care hospital in Montréal, Canada, between 2013 and 2017, was taking a gabapentinoid prior to admission [8]. Only a minority of users (17%) had a clear FDA-approved indication. The most common off-label indications included non-diabetic neuropathic pain (22%), musculoskeletal pain (16%) and cancer-associated pain (9%). Gabapentinoid users were more comorbid than non-users and were more

likely to be co-prescribed opioids (28% of users vs. 12% of non-users) and benzodiazepines (25% of users vs. 14% of non-users). This is very concerning in light of the evidence of increased opioid-related death with co-prescription of either gabapentin [7] or pregabalin [6].

The high prevalence of off-label use of gabapentinoids in comorbid patients who are vulnerable to their common side effects makes this class of drug an ideal target for deprescription. Deprescribing can be defined as the "systematic process of identifying and discontinuing drugs in instances in which existing or potential harms outweigh existing or potential benefits within the context of an individual patient's care goals, current level of functioning, life expectancy, values, and preferences" [23]. There exist many barriers for engagement of patients in deprescription, such as lack of support from a healthcare provider, misinformation regarding the risks and expected benefits of medications, perceived poor health, ongoing active medical issues and fear of withdrawal symptoms [24]. Barriers for physicians include prioritization of the management of acute medical illnesses over deprescribing, especially in an inpatient setting [25]. A reflection of this is the low deprescription rate of gabapentinoids in the previously mentioned study of medical inpatients, with only 13% of cessation or taper at discharge with intention to stop [8].

Multiple strategies exist to improve deprescription of potentially inappropriate medications. One promising method includes the use of direct patient education through the distribution of brochures [26]. These educational brochures contain self-assessments of knowledge of risks of medications, provide information pertaining to the evidence and potential harms of medications, present safer pharmacological and non-pharmacological alternatives for treatment and propose tapering regimens through the use of illustrated calendars, with instructions to contact a designated healthcare provider prior to discontinuing or tapering the medication. The goal of these pamphlets is to improve successful deprescription by promoting patient motivation and self-capacity, as well as soliciting opportunity for deprescription by encouraging patients to meet with their healthcare providers [24]. Specific brochures exist for different classes of drugs, such as benzodiazepines, opioids, gabapentinoids and proton pump inhibitors. Examples of brochures are available on the Canadian Deprescribing Network website at https://www.deprescribingnetwork.ca/patient-handouts. The distribution of brochures can take place in different contexts of patient care. In the EMPOWER trial (Eliminating Medications Through Patient Ownership of End Results), 303 community-dwelling older adults who were chronically taking benzodiazepines were randomly assigned to receiving either an educational brochure by mail or usual care [26]. At 6 months of follow-up, 27% of the intervention group had discontinued their benzodiazepine, versus 5% in the control group (number needed to treat 4).

The promising results of the EMPOWER trial inspired a subsequent study of sedative deprescription with the use educational brochures in medical inpatients [9]. Older adults admitted to a medical ward of a tertiary-care hospital in Montreal, Canada, were screened for chronic sedative use. A total of 50 patients received an educational brochure while hospitalized. At 30 days post-discharge, 64% of those patients had discontinued their sedative, which was significantly higher than the historical deprescription rate of 21% (number needed to treat 3). The higher deprescription rate associated with the intervention in this study (64%) compared to the EMPOWER trial (27%) illustrates the importance of seizing hospital admissions as an opportunity for deprescription. These events can drive patient motivation for change, particularly when presenting symptoms are related to side effects of the offending drug (for example, falls in a benzodiazepine user). Furthermore, the proximity of medical staff on the ward makes it easier for patients to discuss deprescription and initiate a tapering plan. Indeed, deprescription was shown to be more successful when initiated during hospitalization, rather than after discharge [9].

## Research design and methodology

This will be a prospective controlled before and after study involving two centres: the McGill University Health Centre (MUHC) Glen Site (Royal Victoria Hospital) and the Montreal General Hospital. The control and intervention units will be the five medical units.

The study will begin in the control period. Based on previous study data regarding the prevalence of admitted gabapentinoid users and the average number of monthly admissions on the clinical teaching units, recruitment in the control period should last approximately 12 months. All study sites will simultaneously transition to the intervention period after 80 patients have been recruited (estimated 1 year). The rationale for simultaneous transition of all units is to reduce the risk of inter-site contamination of the intervention, as it is common for medical residents and staff to work in all the study sites during a given year. The decision to transition to the intervention period after 80 patients have been recruited is motivated by the need to reduce the risk of bias introduced by temporal trends in deprescribing secondary to level of training of junior residents and seasonal variations of workload on the medical wards. If the target number of control participants is reached before the 12-month mark, recruitment will be suspended until then. The intervention period will be completed once the pre-specified target number of 80 intervention participants is reached, which is expected to take approximately 12 months as well.

Follow up of discharged patients will be at 8 weeks post-discharge. We will analyze and publish the results afterwards.

## Controls

All units will serve as their own controls during the initial control period. During this period, participants admitted to the medical ward with gabapentinoid use pre-admission (which are referred to as "gabapentinoid users") will receive usual medical care (pharmacy medication reconciliation). Although they will be informed that the goal of the trial is to evaluate medications and changes post discharge, they will be unaware that gabapentinoids are specifically targeted, so as to reduce the risk of contamination of the control arm. Furthermore, the medical staff will not receive specific information about the trial, or particular instructions with regards to deprescription during the control period.

## The intervention

The intervention will include the following components:

***1. In-hospital patient educational brochure:***

Participant's medication list will be identified from the pharmacy database and validated with the participant's best possible medication history taken at time of admission by the pharmacist. Eligibility will be confirmed by the participant's treating medical team with support from the study investigators. Participants who are enrolled during the intervention phase will receive an educational brochure (see appendix) about the risks of chronic gabapentinoid use and how to stop using them. The rationale for the structure of the brochure has been presented in detail [24] and it has been validated in both English and French for comprehension and readability, including for people with mild cognitive impairment [27].

***2. Educational session toward physicians about gabapentinoid prescriptions:***

An educational session about the purpose of the study and risks of gabapentinoid prescriptions will be delivered to physicians (staff and medical residents) on each unit at both sites during the intervention period. The educational session will include a presentation by a member of the team at the monthly teaching rounds and an electronic message sent to all physicians on the medical service. They will also be presented a brief overview of the educational brochures.

## Participant selection and recruitment

Any patient aged 60 years or older that has a prescription for gabapentin or pregabalin will be evaluated for the study by a research assistant. The 60 year cutoff was chosen based on retrospective data from the MUHC which identified that 75% of gabapentinoid users admitted to the medical CTU were aged 60 years and older. [8] Furthermore, given the increased prevalence of polypharmacy in older adults and its association with higher risks of adverse drug effects, this population would benefit most from

strategies to improve deprescription of potentially inappropriate medications. Patients will be screened with automated reports of active medications of inpatients provided by the institutional pharmacy. Pre-admission use of gabapentinoids will be confirmed with the medication history obtained by the unit's pharmacist at time of admission. If medication history is not available, then the admission note of the medical team will be used for confirmation. A research assistant will meet with members of the medical team to inform them of the eligibility of subjects for the study. A member of the medical team will then present the study to these potential participants and inquire if they would be interested in receiving further information. If so, they will subsequently be met by the research assistant.

**Inclusion and exclusion criteria**

*Inclusion criteria:*

- All inpatients 60 years or older admitted to study units who have a gabapentinoid prescription pre-admission. The 60-year cut-off was selected as it was felt this group would benefit the most from a deprescribing intervention, given the higher prevalence of polypharmacy and risk for potential adverse drug events. The deprescribing brochures are also designed with the input of older adults and specifically address changes in physiology that accompany the aging process to underscore the risks of gabapentinoids. Based on published retrospective data from the MUHC, approximately 75% of admitted gabapentinoid users are aged 60 and older [8].

*Exclusion criteria:*

- Less than 60 years of age
- Known seizure disorder
- Not being enrolled in the provincial drug plan (RAMQ)
- Opting out of the provincial drug database (*Dossier Santé Québec*), which will be confirmed by the patient at time of recruitment
- Previous enrollment in the study
- Patients expected to die before primary endpoint can be realized (e.g., patients admitted for end-of-life care or prognosis of 3 months or less)
- Unable to consent
- Major neurocognitive disorder, as determined by a previously established diagnosis or interim diagnosis by the medical staff on the ward of moderate severity or worse.
- Inability for patient to speak English or French
- Insufficient literacy in English or French
- No means of contacting patient by phone after discharge (for example, no valid phone number, traveling or planned discharge to facility without means of reaching by phone, etc.)

**Justification of sample size**

The estimated sample size is 160 participants, with 80 in the control group and 80 in the intervention group. The sample size was calculated to detect an absolute increase of 20% in deprescription rates with the intervention (number needed to treat 5), allowing for a two-sided type 1 error of 5% and a type 2 error of 20%. Based on the results of a previous retrospective descriptive study of gabapentinoid prescriptions in medical inpatients, the control period event rate (discontinuation) is expected to be 13% [8]. The expected loss to follow-up is approximately 15%, including deaths.

There are approximately 50 patients admitted per month on each clinical teaching unit (total 200 admissions across the study units), of which 13% are expected to be taking gabapentinoids prior to admission [8]. Approximately 75% of admitted gabapentinoid users are aged 60 years and older. Assuming a refusal rate of 15%, as seen in the EMPOWER study [26], we expect a maximum of 16 participants enrolled per month.

**Data collection**

Information about participants will be collected by the research assistants from the participants and their medical record. We will abstract reason for admission, comorbidities and other medications from the chart. At time of enrollment, we will obtain consent to directly obtain prescription history from the *Dossier Santé Québec* (DSQ), which is a prescription record database for all patients in Québec, as well as from the community pharmacy from which participants receive their medications. These will serve to validate deprescription. During the index admission, a first questionnaire will be administered in person and will collect information about demographics, including age, gender and residence style (independent, assisted living, etc.). We will also obtain a medication use history (for study drugs) and standardized questionnaires to assess global functioning, pain control and cognition. Follow up questionnaires will be administered at 8 weeks after hospital discharge by telephone. The follow-up questionnaire will collect any demographic changes such as destination of discharge and length of stay, will ask participants about deprescription (cessation, reduced use, intent to reduce, or no change), initiation or dose increase of other pain medications, occurrence of withdrawal symptoms, and finally will repeat the global functioning, pain control and cognition questionnaires.

Death will be determined from hospital records. Participants who are known to have died before 8 weeks post-discharge will be excluded from the analysis of the primary outcome (as they cannot attain it); however, they will be retained in the study for descriptive purposes and other outcomes.

**Language**

All materials will be available in English and French.

**Protecting against sources of bias**

*Contamination between intervention and control:*

This will be a controlled before and after study, with simultaneous crossover of all study units into the intervention period. Allocation of the intervention in a before and after fashion will avoid contamination between units, as medical staff might be enticed to increase deprescription for all admitted gabapentinoid users once the educational brochures are handed out, regardless of the intervention group of the participants. **Inter-site** contamination between intervention and control groups is limited by the simultaneous crossover of all sites. Indeed, it is common for medical staff and residents to rotate at all study sites during a given year. If we were to have different units simultaneously in the control and intervention periods, we could expect a source of contamination by having medical staff rotate from an intervention site to a control site afterwards.

At each hospital, it is possible that a participant will be admitted prior to the transition from control to intervention. Participants enrolled during the control period who remain inpatients when the site is switched over to the intervention period will remain in the control arm.

*Blinding:*

It will not be possible to blind participants or physicians in this study. However, this is typical in pragmatic study designs, and we do not expect that this will have a substantial impact on our results. Blinding the assessment of outcome will be critical to ensure valid study results and we have tried to select as many hard outcomes as possible, including validated standardized questionnaires, that do not involve individual judgement by the assessor.

In the control period of this study, participants will be informed that the study will be evaluating medications and post-discharge changes but will not be aware that gabapentinoids will be specifically studied, in order to reduce selection bias. Accordingly, medical staff will not be aware of the specifics of the study during the control period.

*Other:*

Given the simultaneous crossover of all sites into the intervention period, our study might be subject to temporal trends in deprescription of gabapentinoids over the year, which could be influenced by

factors such as training level of junior residents or time-constraints from seasonal variations of workload intensity on the clinical teaching unit. However, previous retrospective data has not shown any temporal trends for gabapentinoid deprescribing on medical wards [8]. The control period and intervention periods will be spaced out such that they will bridge the academic year as best as possible.

**Quality Control / Quality Assurance**

Ongoing quality control and quality assurance will be reviewed in regular research team meetings throughout this study to identify any concerns about the implementation of the intervention, data collection and/or analysis.

**Ethical considerations, consent, privacy and confidentiality**

The study will be conducted in compliance with TCPS 2 (2018), as well as in respect of the requirements set out in the applicable standard operation procedures of the Research Institute of the McGill University Health Centre and of the McGill University Health Centre Research Ethics Board. Ethics approval will be obtained through the McGill University Health Centre Research Ethics Board.

Participant information will be compiled using the MUHC Research Institute REDCap software. Data will be coded with the use of an unique code for each participant. Access to the database will be limited to the study investigators and research assistants and will require a two-factor authentication. Only study investigators will have access to the final study data.

Some subjects may only agree to participate in the study if they are exempt from the follow up phone calls.  Such patients will still be enrolled if they consent to our use of the DSQ to evaluate the primary endpoint but will be excluded from some of the secondary analyses.

Patients who are cognitively impaired or otherwise unable to provide consent will not be included. Although this subpopulation of patients may still greatly benefit from deprescription of potentially inappropriate medications, the educational brochures used in this study rely on instigating motivation and providing self-capacity, and are therefore intended for patients who are cognitively intact or have only a mild form of cognitive impairment [27]. Although distribution of the educational brochures to the patient's proxy could be considered, this strategy is less feasible in the context of the SARS-CoV-2 pandemic, as family and caregivers have limited access to the bedside, hindering contact with medical staff, which is crucial for initiation of the deprescription process.

Patients who are ultimately transferred to and discharged from non-study units during their hospitalization will be excluded unless that unit is a transitional care, rehabilitation, or post-acute care unit used only to bridge the gap between acute medical hospitalization and community services.

**Data analysis and evaluation of results**

STATA (StataCorp LP, College Station, Texas) will be used for the analysis.

**1. Develop benchmark data for inappropriate medication use that can be compared across hospitals in Quebec and Canada.**

We will provide a descriptive summary of the population enrolled in this study including participant age, gender, comorbidities, type of prescription, other prescriptions, length of stay, residence type, and the number who enrolled in deprescription. (i.e., Table 1).

**2.Evaluate the effectiveness of a hospital-based deprescription intervention for unnecessary gabapentinoid prescriptions.**

Participants will be grouped by control and intervention based on the timing of the study. The primary outcome will be the complete cessation of gabapentinoid use or intent to taper to discontinuation (prescribed taper) measured at 8-weeks post hospital discharge. A secondary outcome will be measured as a dose reduction, without any intent to taper to discontinuation (decrease in medication use after

exposure to the intervention but no taper to complete cessation prescribed at the time of the 8-week study follow-up period).

**Statistical analysis plan**

We will use an "intention to treat" principle. We will analyze participant data according to their participation during the intervention or control period. For the primary outcome, we will perform a sensitivity analysis for patients who died or were lost to follow-up using the most recent available data on their gabapentinoid use (inpatient pharmacy records, discharge prescriptions or the provincial electronic medical record). We will assess binary outcomes using binomial regression comparing the intervention to no intervention and adjusting for age and sex. We will assess continuous outcomes (T scores) using linear regression comparing the intervention to no intervention, adjusting for baseline values, age and sex.

We will perform several preplanned subgroup analyses for the primary outcome, including an analysis of participants aged 80 or older; analyses based on reason for admission, discharge destination (community v. long-term care facility) and comorbidities; and an analysis looking at the effect of each study unit. Subgroup analyses will be treated as hypothesis generating and presented graphically with 95% confidence intervals.

**Project duration**

This ~24-36 month (12 months for control period, 12 months for intervention period, 8 weeks of follow-up post-discharge) trial is scheduled to begin in March 2021 and end in April 2023.

**Dissemination plans**

Results of this study will be disseminated through the publication in a peer-reviewed journal, as well as through presentations in national or international conferences.

**Project risk assessment**

| Risk | Probability | Impact | Mitigation strategy |
|---|---|---|---|
| Describe | high/medium/low | high/medium/low | How do you plan to mitigate the risk? |
| PI time | Low | High | The successful execution of this study depends on sufficient time from the study PI, Dr. Emily McDonald. She has allocated 0.1 FTE in order to have sufficient time to lead this study. The study is supported by a junior investigator (Dr. Gingras), who will lead the project for his fellowship scholarly research activity.<br><br>Dr. McDonald has completed several large RCTs (including a 6000+ patient multicentred deprescribing study) and is well established as an international leader in deprescribing research. |
| Withdrawal symptoms | Low | High | Some participants may experience withdrawal symptoms from reducing gabapentinoid use. There is a low probability of harm to participant health with appropriately prescribed and very |

| | | | |
|---|---|---|---|
| | | | slow tapering. All participants will be followed with usual standard of care to ensure that there is no negative impact on participant well-being associated with the study. Participants will be able to contact Drs. McDonald and Gingras if they are having problems. We believe this risk is mitigated by the potential benefits. Significant harms were not seen in the EMPOWER study [26] nor in the benzodiazepine study conducted at MUHC [9]. |
| Enrolment Problem | Low to medium | High | If we have difficulty enrolling subjects due to ineligibility or refusal, this would have significant risk to create an underpowered study. To mitigate against this we have been very conservative (biasing against the study) in our estimates for sample size. |

## Researcher qualifications

**Emily McDonald,** MD MSc is an Assistant Professor of Medicine and Scientist at the RI-MUHC as well as the Director of the Clinical Practice Assessment Unit. She completed an MSc Epidemiology at McGill University and has numerous publications in top medical journals on a variety of patient safety topics. Dr. McDonald is the Associate Chair of Quality and Safety and works extensively at all MUHC sites involved in the study. She is an established clinical trialist with extensive experience in medication safety randomized controlled trials.

**Todd C. Lee,** MD MPH is an Associate Professor of Medicine. In 2015, his work has been recognized with the Canadian Society of Internal Medicine's *New Investigator Award*. He is an established clinical trialist with extensive experience in medication safety randomized controlled trials.

**Marc-Alexandre Gingras**, MD MSc is a fourth-year resident in General Internal Medicine at McGill University. He has completed an MSc in Biomedical Sciences at Université de Montréal. His research interests include polypharmacy and strategies for improving deprescription. He has previously published in the field of overprescribing gabapentinoids and performed a large retrospective study related to this topic at the MUHC.

## Conflict of interests

Dr. Emily McDonald and Dr. Todd Lee are the co-owners of MedSafer, an electronic deprescribing software for older adults with polypharmacy (it will not be used in the current study).

They both have research salary support from the FRQS

They have public funding from the CIHR, CABHI, CFN and MI4 for clinical trials unrelated to the current study.

No industry support to declare or any other potential conflicts of interest for the current study.

Dr. Marc-Alexandre Gingras has no conflict of interest to declare.

## Appendix

- Appendix 1: References

**Appendix 1:**

**References:**


1. U.S. Food and Drug Administration. *LYRICA - Highlights of Prescribing Information*. 2012; Available from: https://www.accessdata.fda.gov/drugsatfda_docs/label/2012/021446s028lbl.pdf.

2. U.S. Food and Drug Administration. *NEURONTIN - Highlights of Prescribing Information*. 2017; Available from: https://www.accessdata.fda.gov/drugsatfda_docs/label/2017/020235s064_020882s047_021129s046lbl.pdf.

3. Pfizer Canada Inc. *Product Monograph - Lyrica*. 2016; Available from: https://www.pfizer.ca/sites/g/files/g10037206/f/201710/LYRICA_DC_PM_198215_6Dec2016_E.pdf.

4. Pfizer Canada Inc. *Product Monograph - Neurontin*. 2018; Available from: https://pdf.hres.ca/dpd_pm/00044022.PDF.

5. Goodman, C.W. and A.S. Brett, *A Clinical Overview of Off-label Use of Gabapentinoid Drugs.* JAMA Intern Med, 2019.

6. Gomes, T., et al., *Pregabalin and the Risk for Opioid-Related Death: A Nested Case-Control Study.* Ann Intern Med, 2018. **169**(10): p. 732-734.

7. Gomes, T., et al., *Gabapentin, opioids, and the risk of opioid-related death: A population-based nested case-control study.* PLoS Med, 2017. **14**(10): p. e1002396.

8. Gingras, M.A., et al., *Retrospective Cohort Study of the Prevalence of Off-label Gabapentinoid Prescriptions in Hospitalized Medical Patients.* J Hosp Med, 2019. **14**(9): p. E1-e4.

9. Wilson, M.G., et al., *EMPOWERing Hospitalized Older Adults to Deprescribe Sedative Hypnotics: A Pilot Study.* J Am Geriatr Soc, 2018. **66**(6): p. 1186-1189.

10. *Medicines use and spending in the U.S. — a review of 2016 and outlook to 2021*. 2017; Available from: https://structurecms-staging-psyclone.netdna-ssl.com/client_assets/dwonk/media/attachments/590c/6aa0/6970/2d2d/4182/0000/590c6aa069702d2d41820000.pdf?1493985952.

11. Bennett, M.I., et al., *Pregabalin for the management of neuropathic pain in adults with cancer: a systematic review of the literature.* Pain Medicine, 2013. **14**(11): p. 1681-8.

12. Mathieson, S., et al., *Trial of Pregabalin for Acute and Chronic Sciatica.* New England Journal of Medicine, 2017. **376**(12): p. 1111-1120.

13. Moore, A., S. Derry, and P. Wiffen, *Gabapentin for Chronic Neuropathic Pain.* Jama, 2018. **319**(8): p. 818-819.

14. Shanthanna, H., et al., *Benefits and safety of gabapentinoids in chronic low back pain: A systematic review and meta-analysis of randomized controlled trials.* PLoS Medicine / Public Library of Science, 2017. **14**(8): p. e1002369.

15. Wiffen, P.J., et al., *Gabapentin for chronic neuropathic pain in adults.* Cochrane Database Syst Rev, 2017. **6**: p. Cd007938.

16. Wiffen, P.J., et al., *Antiepileptic drugs for neuropathic pain and fibromyalgia - an overview of Cochrane reviews.* Cochrane Database of Systematic Reviews, 2013(11): p. CD010567.

17. Jette, N., et al., *Association of antiepileptic drugs with nontraumatic fractures: a population-based analysis.* Arch Neurol, 2011. **68**(1): p. 107-12.

18. Toth, C., *Drug safety evaluation of pregabalin.* Expert Opinion on Drug Safety, 2012. **11**(3): p. 487-502.

19. Evoy, K.E., M.D. Morrison, and S.R. Saklad, *Abuse and Misuse of Pregabalin and Gabapentin.* Drugs, 2017. **77**(4): p. 403-426.

20. Johansen, M.E., *Gabapentinoid Use in the United States 2002 Through 2015.* JAMA Intern Med, 2018. **178**(2): p. 292-294.

21. Kwok, H., et al., *Impact of Unrestricted Access to Pregabalin on the Use of Opioids and Other CNS-Active Medications: A Cross-Sectional Time Series Analysis.* Pain Med, 2017. **18**(6): p. 1019-1026.

22. Hamer, A.M., et al., *Gabapentin use in a managed medicaid population.* J Manag Care Pharm, 2002. **8**(4): p. 266-71.

23. Scott, I.A., et al., *Reducing inappropriate polypharmacy: the process of deprescribing.* JAMA Intern Med, 2015. **175**(5): p. 827-34.

24. Martin, P. and C. Tannenbaum, *A realist evaluation of patients' decisions to deprescribe in the EMPOWER trial.* BMJ Open, 2017. **7**(4): p. e015959.

25. Scott, S., et al., *Development of a hospital deprescribing implementation framework: A focus group study with geriatricians and pharmacists.* Age & Ageing, 2019. **49**(1): p. 102-110.

26. Tannenbaum, C., et al., *Reduction of inappropriate benzodiazepine prescriptions among older adults through direct patient education: the EMPOWER cluster randomized trial.* JAMA Intern Med, 2014. **174**(6): p. 890-8.

27. Martin, P. and C. Tannenbaum, *Use of the EMPOWER brochure to deprescribe sedative-hypnotic drugs in older adults with mild cognitive impairment.* BMC Geriatr, 2017. **17**(1): p. 37.
