# Supplementary material for: Direct-to-Consumer Educational Brochures to Promote Gabapentinoid Deprescribing in Older Adults
Source: JAMA Intern Med. 2024 Sep 23;184(11):1386–8. doi: 10.1001/jamainternmed.2024.4748 (PMC11420817; doi:10.1001/jamainternmed.2024.4748)
Supplement: Supplement 3. — Data sharing statement [file jamainternmed-e244748-s003.pdf]

Data Sharing Statement

Gingras. Direct-to-Consumer Educational Brochures to Promote Gabapentinoid Deprescribing in Older Adults. *JAMA Intern Med*. Published September 23, 2024.
doi:10.1001/jamainternmed.2024.4748

Data
Data available: Yes
Data types: Deidentified participant data
How to access data: Plese contact todd.lee@mcgill.ca for deidentified patient data
When available: beginning date: 09-01-2025, end date: 09-01-2027

Supporting Documents
Document types: None

Additional Information
Who can access the data: Academics requesting access to the data for secondary analysis whose proposal for the data has been approved by Todd Lee and with a data sharing agreement in place.
Types of analyses: Secondary analysis for academics (non-industry requests)
Mechanisms of data availability: After approval of a proposal and with a signed data sharing agreement
Any additional restrictions: Not for use by members of industry
